# Scleroderma myocarditis with severe secondary mitral regurgitation successfully treated with transcatheter edge-to-edge repair: a case report

**DOI:** 10.1093/ehjcr/ytae425

**Published:** 2024-08-13

**Authors:** Daisuke Sato, Tomoki Ochiai, Takashi Matsumoto, Shingo Mizuno, Shigeru Saito

**Affiliations:** Department of Cardiology, Shonan Kamakura General Hospital, 1370-1, Okamoto, Kamakura City, Kanagawa 247-8533, Japan; Department of Cardiology, Shonan Kamakura General Hospital, 1370-1, Okamoto, Kamakura City, Kanagawa 247-8533, Japan; Department of Cardiology, Shonan Kamakura General Hospital, 1370-1, Okamoto, Kamakura City, Kanagawa 247-8533, Japan; Department of Cardiology, Shonan Kamakura General Hospital, 1370-1, Okamoto, Kamakura City, Kanagawa 247-8533, Japan; Department of Cardiology, Shonan Kamakura General Hospital, 1370-1, Okamoto, Kamakura City, Kanagawa 247-8533, Japan

**Keywords:** Mitral transcatheter edge-to-edge repair, Mitral regurgitation, Myocarditis, Scleroderma, Case report

## Abstract

**Background:**

Systemic sclerosis presents with a variety of cardiac manifestations, while myocarditis is usually a rare finding. Furthermore, there are no reports on the use of mitral transcatheter edge-to-edge repair (M-TEER) for the treatment of severe ventricular functional mitral regurgitation (vFMR) secondary to scleroderma myocarditis.

**Case summary:**

A-79-year-old male was admitted to our hospital because of fever and fatigue. His physical examination revealed thickening of the fingertips’ skin, Raynaud phenomenon, and mild pedal oedema. Positive anti-centromere antibodies indicated a diagnosis of a limited cutaneous systemic sclerosis. He presented with symptoms of heart failure, and moderate to severe lymphocytic infiltration was evident in his endomyocardial biopsy. He responded well to medical therapy and was discharged. However, one month after hospital discharge, he was readmitted to our institution because of worsening heart failure. Transthoracic echocardiography showed a decrease in left ventricular systolic function and progression of left ventricular remodelling, which caused severe vFMR. Endomyocardial biopsy revealed decreased lymphocytic infiltration and mild myocardial interstitial fibrosis, indicative of scleroderma myocarditis. As he was unable to be weaned off inotropes, we performed M-TEER for severe vFMR, which led to a significant reduction in MR volume and improvement of heart failure symptoms. A week after procedure, immunosuppressive therapy was initiated and the patient was discharged home in stable condition.

**Discussion:**

Scleroderma myocarditis may manifest as heart failure with reduced ejection fraction with severe vFMR. Mitral transcatheter edge-to-edge repair for severe vFMR in the context of myocarditis can be one of the therapeutic options for haemodynamic stabilization.

**Learning points**

- Scleroderma myocarditis may present with severe ventricular functional mitral regurgitation (vFMR) secondary to heart failure with reduced ejection fraction.
- Mitral transcatheter edge-to-edge repair (M-TEER) is expected to provide rapid haemodynamic stabilization by reducing severe vFMR.
- The combination of M-TEER with immunosuppressive therapy can be a viable therapeutic option for patients with scleroderma myocarditis and severe vFMR.

## Introduction

In patients with chronic severe secondary mitral regurgitation (MR) related to left ventricular systolic dysfunction who remain symptomatic despite optimal guideline-directed management and therapy (GDMT) for heart failure, mitral transcatheter edge-to-edge repair (M-TEER) has been shown to reduce mortality and heart failure hospitalizations.^1^ Furthermore, it can be a potentially effective treatment of severe MR in patients with haemodynamic instability.^2^ Scleroderma myocarditis is a rare cardiac involvement in systemic sclerosis (SSc) and is associated with a poor prognosis.^3^ To date, effectiveness of valvular interventions in patients with scleroderma myocarditis and severe ventricular functional mitral regurgitation (vFMR) remains unexplored. Here, we present a case of haemodynamically unstable patient with scleroderma myocarditis and vFMR that was successfully treated with M-TEER.

## Summary figure

**Table:** 

Day 1	He was admitted to our hospital with fever and fatigue. Transthoracic echocardiography (TTE) showed reduced left ventricle ejection fraction (LVEF) of 35 % with global hypokinesis, mild pericardial effusion, and mild mitral regurgitation (MR).
Day 7	Right ventricular endomyocardial biopsy (EMB) revealed moderate to severe lymphocytic inflammatory infiltrates with no fibrotic changes, eosinophil infiltration, or vasculitis, which suggested acute myocarditis.
Day 35	He was discharged in stable condition.
Day 62	He was readmitted to our hospital due to worsening heart failure. TTE revealed a decrease LVEF, moderate pericardial effusion, and severe ventricular functional MR (vFMR).
Day 70	In addition to optimal guideline-directed medical therapy for heart failure, dobutamine infusion was initiated to optimize haemodynamic status.
Day 85	Right ventricular EMB revealed decreased lymphocytic infiltration and the progression of myocardial interstitial fibrosis.
Day 93	He underwent mitral transcatheter edge-to-edge repair, and one XTW MitraClip was deployed at the middle segment of the anterior and posterior leaflet, leading to significant reduction in vFMR.
Day 104	Immunosuppressive therapy was initiated.
Day 117	He was discharged in stable condition.
Day 209	He was in stable condition and symptomatically improved.

## Case presentation

**Figure 1:**
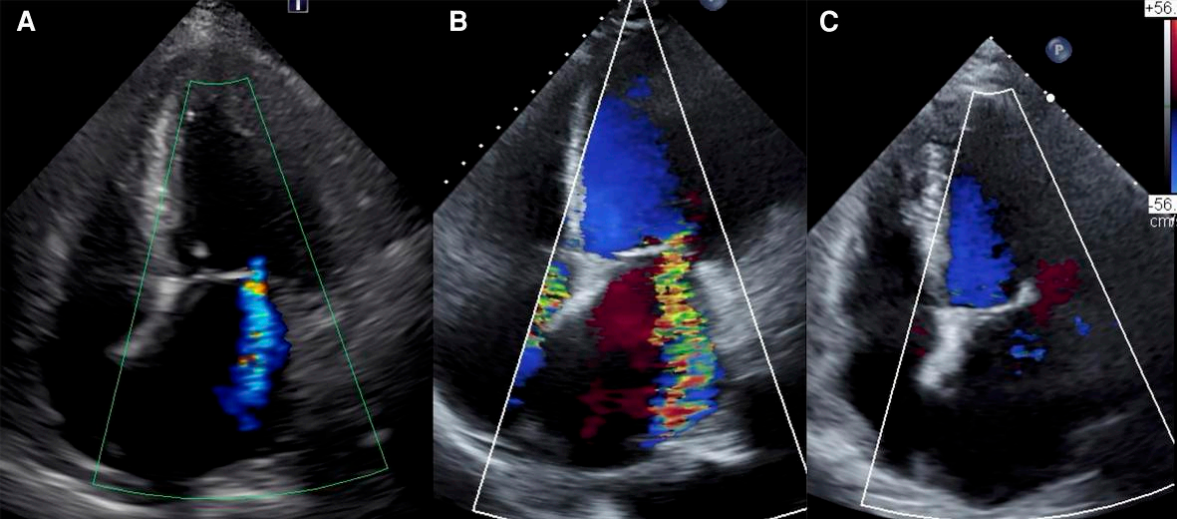
Echocardiographic findings. (*A*) Transthoracic echocardiography (TTE) at 1st admission to our hospital revealed left ventricular ejection fraction (LVEF) of 35% and mild mitral regurgitation (MR). (*B*) TTE before mitral transcatheter edge-to-edge repair of mitral valve (M-TEER) procedure revealed LVEF of 33% and severe MR. (*C*) TTE 3 months after M-TEER revealed LVEF of 44% and mild MR.

**Figure 2:**
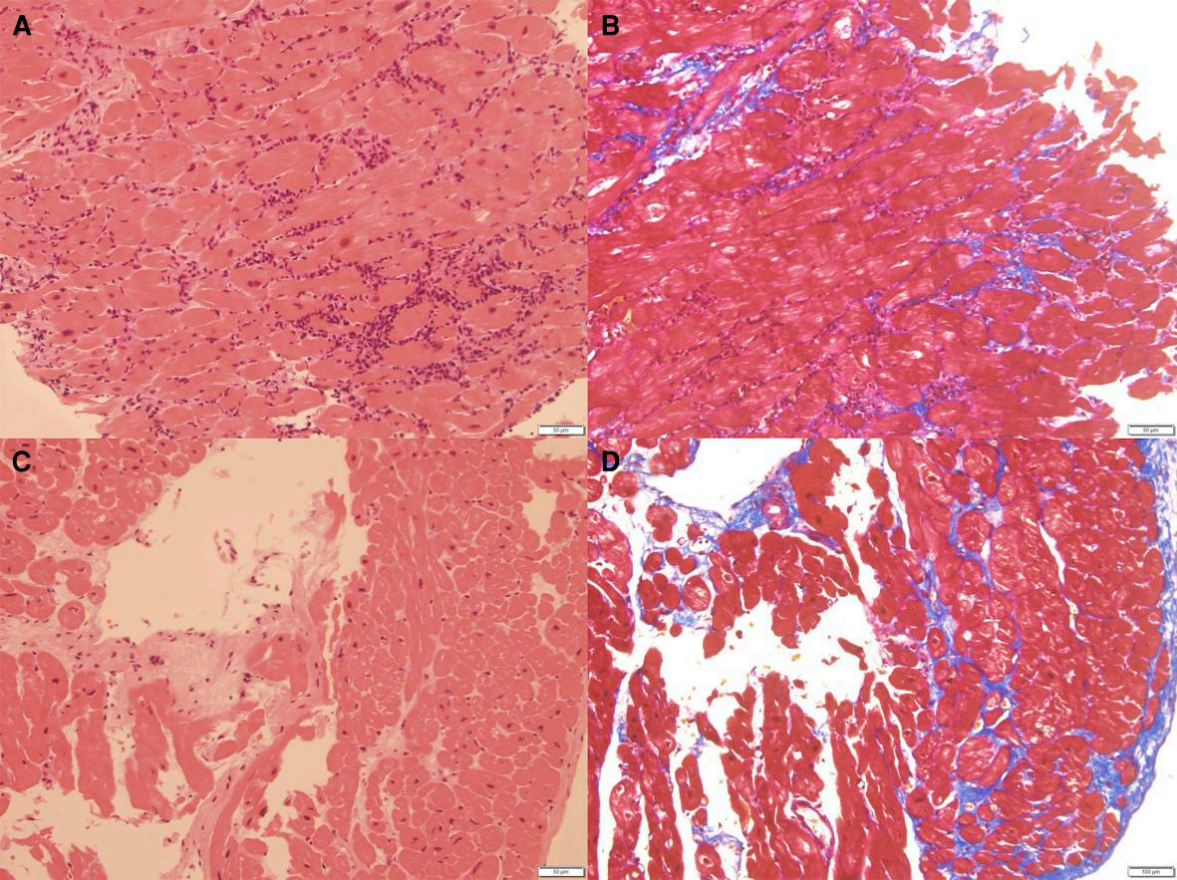
Histopathological findings of the right ventricular septum biopsy. (*A*) Moderate to severe lymphocytic inflammatory infiltrates without fibrotic changes or vasculitis in haematoxylin and eosin staining (Day 7). (*B*) Masson-trichrome staining. (*C*) Decreased lymphocytic infiltration and the progression of myocardial interstitial fibrosis in haematoxylin and eosin staining (Day 85). (*D*) Masson-trichrome staining. Every bar located at the right below the area in every figure indicates 50 μm (*A–D*).

A-79-year-old man was admitted to our hospital with fever and fatigue. His past medical history included hypertension. On his admission, initial vital signs were as follows: blood pressure 179/86 mmHg, heart rate 105 b.p.m., respiratory rate 25/min, and oxygen saturation 94% in room air. He had a thickening of the fingertips’ skin proximal to metacarpophalangeal joints, nail fold bleeding, capillary dilation, Raynaud phenomenon, and mild bilateral pedal oedema. The modified Rodnan skin score was 16. Electrocardiogram revealed atrial fibrillation and left ventricular strain pattern in V3–V6. Laboratory tests revealed elevated levels of cardiac troponin I (8812 pg/mL; normal range < 45.2 pg/mL), serum creatine (3.80 mg/dL; normal range < 1.07 mg/dL), C-reactive protein (22.5 mg/dL; normal range < 0.14 mg/dL), brain natriuretic peptide (BNP) (5077 pg/mL; normal range < 18.4 pg/mL), and creatine phosphokinase (1987 U/L; normal range < 248 U/L). Anti-nuclear antibodies and anti-centromere antibodies were positive but anti-Scl-70 antibodies were negative. Transthoracic echocardiography (TTE) revealed reduced left ventricle ejection fraction (LVEF) of 35% with global hypokinesis, LAVI of 96 mL/m^2^, mild pericardial effusion, and mild MR (*Figure 1A*, Supplement material online, *Video S1*). Right ventricular endomyocardial biopsy (EMB) revealed moderate to severe lymphocytic inflammatory infiltrates with no evidence of fibrotic changes, eosinophil infiltration, or vasculitis, which suggested acute myocarditis (*Figure 2A*). Screening for viral infections was negative. He met the ACR/EULAR criteria for the diagnosis of SSc.^4^ He responded well to GDMT, leading to an improvement of LVEF and symptoms. Thus, immunosuppressive therapy for SSc was deferred and he was discharged in stable condition on Day 35. Medications on discharge included furosemide 40 mg daily and carvedilol 1.25 mg daily.

However, one month later, he was readmitted to our hospital due to worsening heart failure. Chest X-ray showed asymmetric pulmonary oedema. Transthoracic echocardiography revealed decreased LVEF of 33% with global hypokinesis, LAVI of 93 mL/m^2^, and moderate pericardial effusion. Severe vFMR was noted with an effective regurgitant orifice area (EROA) of 0.47 cm^2^, regurgitant volume of 70 mL (*Figure 1B*, Supplement material online, *Video S2*). Doppler-derived pulmonary artery systolic pressure was 58 mmHg. There was mild aortic regurgitation and severe tricuspid regurgitation. Cardiac T2-weighted magnetic resonance imaging revealed a slightly high signal intensity in the mid-layer myocardium with a mild pericardial effusion, supporting the diagnosis of myocarditis. Myocardial perfusion scintigraphy revealed no myocardial ischaemia. Right heart catheterization revealed a mean pulmonary capillary wedge pressure of 24 mmHg, mean pulmonary artery pressure of 34 mmHg, cardiac index of 2.7 L/min/m^2^, and stroke volume index of 32.5 mL/m^2^. Right ventricular EMB revealed decreased lymphocytic infiltration and the progression of myocardial interstitial fibrosis (*Figure 2B*). Based on these results and clinical course, he was diagnosed with scleroderma myocarditis.

**Figure 3:**
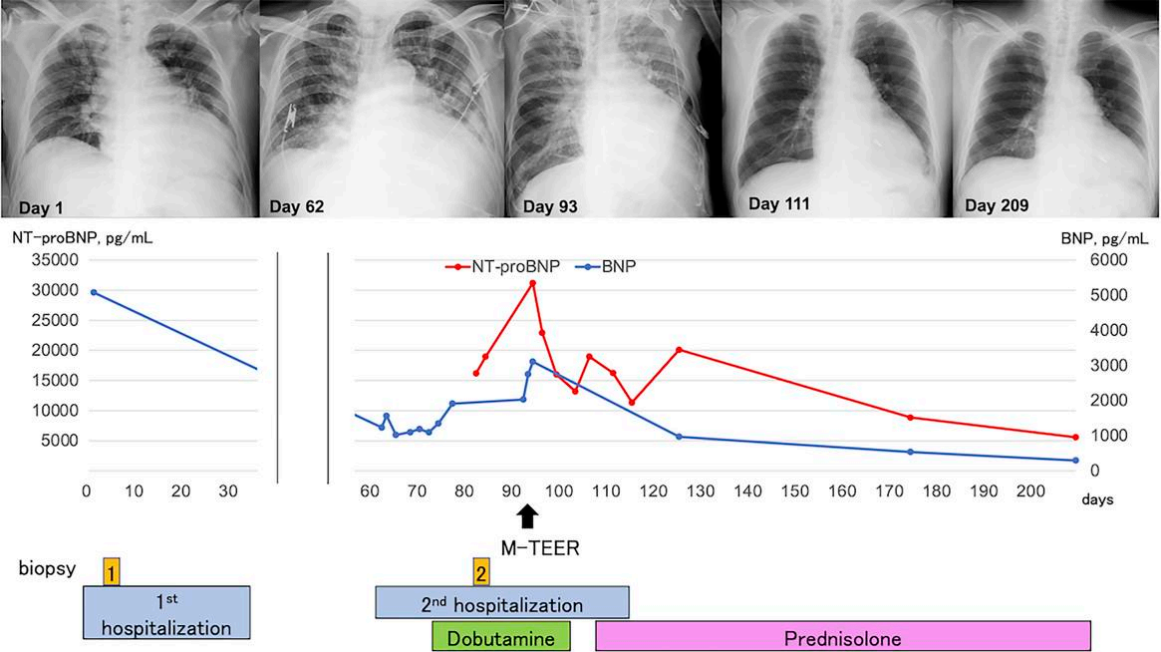
Timeline of the case showing left ventricular ejection fraction, brain natriuretic protein (BNP), and N-terminal pro-BNP (NT-proBNP) from the onset of symptom to 3-month follow-up after discharge. The timing and duration of intravenous dobutamine infusion, immunosuppressive therapies, endomyocardial biopsies, M-TEER, and hospitalizations are shown.

His clinical course is shown in *Figure 3*. He was managed with dapagliflozin 10 mg, bisoprolol 5 mg, tolvaptan 7.5 mg, spironolactone 25 mg, and sacubitril/valsartan 100 mg. In addition to these medications, dobutamine infusion was initiated to optimize haemodynamic status. However, the patient could not be weaned off dobutamine because of persistent low cardiac output. Serum lactate levels were 2 mmol/L (normal range: <1.3 mmoL/L). Systolic blood pressure was 90 mmHg with the inotrope. The level of N-terminal pro-BNP (NT-proBNP) was elevated at 31 193.3 pg/mL (normal range < 125 pg/mL). We considered immunosuppressive therapy for scleroderma myocarditis as the cornerstone of treatment, however, there was concern that the haemodynamic status might not improve despite the initiation of immunosuppressants. Mitral transcatheter edge-to-edge repair was expected to provide relatively rapid haemodynamic stabilization by mitigating FMR and can be a bridge to safe initiation of immunosuppressive therapy. Hence, we prioritized haemodynamic stabilization by reducing FMR with M-TEER before starting immunosuppressive therapy. Consequently, the decision was made to proceed with M-TEER using MitraClip G4 system (Abbott, Menlo Park, CA). He underwent M-TEER, and one XTW MitraClip was deployed on the A2 and P2 scallops (see Supplement material online, *Videos S3–S6*). This resulted in a significant reduction in MR grade from severe to mild with EROA of 0.10 cm^2^. The post-procedural mean transmitral pressure gradient was 2.4 mmHg with mitral valve area of 2.25 cm^2^. After the procedure, there was a remarkable improvement in haemodynamics, and the patient was weaned off dobutamine within the next day. A week after the procedure, TTE revealed the LVEF of 34% with mild MR and mild pericardial effusion. Oral prednisolone 60 mg daily was introduced subsequently, with a gradual dose reduction of 10 mg every other week. The NT-proBNP level decreased to 13 181.8 pg/mL, and TTE revealed a recovery of LVEF of 44%. He was discharged home 25 days after TEER procedure. Medications on discharge comprised dapagliflozin 10 mg daily, bisoprolol 5 mg daily, tolvaptan 7.5 mg daily, furosemide 40 mg daily, spironolactone 50 mg daily, and sacubitril/valsartan 100 mg daily. At 3-month follow-up, he was in stable condition and symptomatically improved. There was a significant reduction in NT-proBNP level to 8964.9 pg/mL. Transthoracic echocardiography revealed LVEF of 44% with mild MR and no pericardial effusion (*Figure 1C*, Supplement material online, *Video S7*).

## Discussion

This case demonstrates M-TEER can be an effective treatment option in haemodynamically unstable patients with severe vFMR secondary to scleroderma myocarditis.

The severity of acute myocarditis varies widely, and the most common clinical problem is a reversible decrease in cardiac function associated with inflammation. In most cases, the inflammatory phase lasts 1–2 weeks, followed by a recovery phase in which cardiac function improves.^5,6^ Therefore, temporal use of inotropes as well as mechanical circulatory support devices such as intra-aortic balloon pump, Impella, or extracorporeal circulatory membrane oxygenation is considered to stabilize haemodynamics especially in the acute phase of myocarditis.

In this case, active myocarditis was indicated based on the Dallas criteria during the first hospitalization. The association of acute myocarditis with SSc remained uncertain at that time, given the improvement in LVEF and symptoms with GDMT. Notably, in cases of acute myocarditis, the routine treatment with immunosuppressive therapy did not yield a significant improvement in LVEF or survival compared to conventional heart failure therapy.^7^ Consequently, we deemed the initiation of immunosuppressive therapy unnecessary during the first hospitalization. However, unlike the typical course of acute myocarditis, this patient presented with worsening heart failure even after surviving the acute phase. The elevated levels of cardiac troponin I and inflammatory markers, a progressive trend towards a decline in LVEF, and the enlargement of the left ventricle over time in TTE, along with the findings from Cardiac T2-weighted magnetic resonance imaging and a right ventricular EMB, collectively supported the diagnosis of scleroderma myocarditis.

Cardiac manifestations are well-recognized complications of SSc and is associated with poor prognosis.^3^ Valvular heart disease was considered rare in SSc apart from functional tricuspid regurgitation associated with pulmonary artery hypertension.^6,8^ However, chronic inflammation of the cardiomyopathies or myocarditis in SSc can lead to LV remodelling, which is a major cause of severe vFMR and unstable haemodynamics.

In this case, it was considered that haemodynamic instability was attributed to severe vFMR associated with scleroderma myocarditis. In patients with chronic severe secondary MR related to LV systolic dysfunction who have persistent symptoms while on optimal GDMT, M-TEER can be a therapeutic option if anatomically suitable.^1^ Furthermore, several retrospective studies showed that M-TEER was safe and effective treatment option in haemodynamically unstable patients and led to a lower rate of hospitalization for heart failure or lower all-cause mortality.^2,9–11^ Therefore, M-TEER was expected to provide relatively rapid haemodynamic stabilization by mitigating FMR and can be a bridge to immunosuppressive therapy. Furthermore, the use of immunosuppressants was associated with infection, vascular complications, or tissue fragility, which may pose challenges to future mitral valve interventions.^12–14^ Hence, we prioritized haemodynamic stabilization by reducing FMR with M-TEER before starting immunosuppressive therapy. Although several cases reported that immunosuppressive therapy was effective for scleroderma myocarditis, optimal treatment strategy for this condition has not been established in large-scale studies.^15^ In addition, it was not anticipated that immunosuppressive therapy would rapidly improve the haemodynamic instability of the patient. The long-term durability of M-TEER remains unclear, however, our case demonstrates the safety and effectiveness of M-TEER in a patient with scleroderma myocarditis and severe vFMR.

## Conclusions

Our findings suggest that M-TEER can be a safe and effective therapeutic option for patients with scleroderma myocarditis and severe vFMR. Further studies are required to establish an appropriate therapeutic strategy in such cases.

## Lead author biography


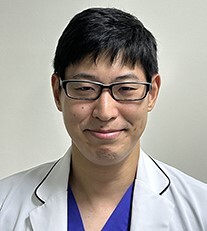


Daisuke Sato was graduated from Tohoku University in 2018. He finished resident programme (2018.4–2020.3) and fellowship in Internal Medicine (2020.4–2023.3). He joined the Department of Cardiology in 2023. He had learned interventional cardiology, ischaemic heart diseases, and structure heart diseases. Membership: Japanese Circulation Society, Japanese Association of Cardiovascular Intervention and Therapeutics.

## Supplementary Material

ytae425_Supplementary_Data

## Data Availability

The data underlying this article are available in the article.

## Notes

**Consent**: The authors confirm that written consent for submission and publication of this case report including images and associated text has been obtained from the patient in line with COPE guidance.

**Funding:** None.
